# Perceived stress related to the Russia–Ukraine war in nine Latin American countries

**DOI:** 10.3389/fpsyg.2026.1845161

**Published:** 2026-06-16

**Authors:** Christian R. Mejia, Tatiana Requena-Pastorelli, Víctor Serna-Alarcón, Jamil Cedillo-Balcázar, Leonel Vega-Perez, Camilo Vega-Useche

**Affiliations:** 1 Universidad de Huánuco, Huánuco, Peru; 2 Universidad Cesar Vallejo, Trujillo, Peru; 3 Universidad Privada Antenor Orrego, Piura, Peru; 4 Hospital de la Amistad Perú Corea Santa Rosa II-2, Piura, Peru; 5 Dirección Provincial de Salud de Loja, Ministerio de Salud Pública, Loja, Ecuador; 6 Instituto Superior Tecnológico Daniel Álvarez Burneo, Loja, Ecuador; 7 Universidad Pedagógica y Tecnológica de Colombia, Tunja, Colombia; 8 Fundación Santa Fe de Bogotá, Bogotá, Colombia

**Keywords:** armed conflicts, Latin America, mental health, psychological stress, war

## Abstract

**Introduction:**

Large-scale geopolitical crises may generate psychological distress beyond populations directly exposed to violence, particularly through uncertainty, perceived lack of control, and indirect exposure to threatening information.

**Objective:**

To determine the association between country of residence and perceived stress related to the Russia–Ukraine war among adults from nine Latin American countries.

**Methods:**

A cross-sectional analytical study was conducted during March and April 2022. Perceived war-related stress was assessed using the validated nine-item War-Stress scale. Three binary outcomes were defined using the empirical upper tercile of the overall score and its two prespecified subcomponents: conflict-specific stress and external-factor stress. Adjusted prevalence ratios were estimated using Poisson regression models with robust variance, controlling for sex, age, and educational level.

**Results:**

The final analytical sample included 2,463 participants. The most frequently endorsed stress-related item was concern that the conflict could escalate into a larger war, with 29.72% agreeing and 19.57% strongly agreeing. In adjusted analyses, Argentina showed a higher prevalence of overall high war-related stress compared with Colombia (aPR: 1.73; 95% CI: 1.33–2.25; *p* < 0.001) and also higher prevalence of high conflict-specific stress (aPR: 1.58; 95% CI: 1.13–2.21; *p* = 0.008). For high external-factor stress, Argentina again showed the strongest association (aPR: 3.03; 95% CI: 2.30–3.99; *p* < 0.001), whereas Peru (aPR: 0.52; 95% CI: 0.38–0.73; *p* < 0.001) and Brazil (aPR: 0.50; 95% CI: 0.28–0.90; *p* = 0.020) showed lower prevalence. Men consistently exhibited lower prevalence of high perceived stress across the three outcomes.

**Conclusion:**

Perceived stress related to the Russia–Ukraine war varied across Latin American countries and was especially associated with external and anticipatory dimensions of the conflict. The findings support the relevance of studying indirect psychological responses to distant geopolitical crises in populations already exposed to broader social and post-pandemic vulnerabilities.

## Introduction

Large-scale geopolitical crises can affect mental health beyond the populations directly exposed to violence, displacement, or material loss. Wars, pandemics, and other collective threats may generate psychological distress in geographically distant populations through indirect exposure, anticipatory uncertainty, perceived lack of control, and repeated contact with threatening information through traditional and digital media. In the context of armed conflicts, this form of vicarious or mediated exposure may trigger stress, anxiety, depressive symptoms, and post-traumatic responses even among individuals who are not physically located in war zones, particularly when the event is perceived as globally consequential or capable of disrupting personal and societal security (Lim et al., 2022; Chudzicka-Czupała et al., 2023; Celuch et al., 2024; Kesner et al., 2025).

This phenomenon can be interpreted through transactional and appraisal-based models of stress. From this perspective, psychological responses are shaped not only by the objective characteristics of a threat, but also by how individuals evaluate its severity, controllability, predictability, and implications for their own lives. Events perceived as escalating, difficult to control, or associated with uncertain future consequences may activate sustained stress responses, especially when coping resources are already strained (Taha et al., 2014; Spătaru et al., 2024). Research on public reactions to global crises supports this interpretation. Greater exposure to threatening media content has been associated with acute stress, anxiety, and depressive symptoms, partly through intolerance of uncertainty, low perceived control, and reduced social support (He et al., 2021; Phalswal et al., 2023). Similarly, during the Russia–Ukraine war, concerns about the conflict were linked to perceived stress and psychological distress in populations outside the immediate war zone (Chudzicka-Czupała et al., 2023; Celuch et al., 2024; Scharbert et al., 2024).

The Russia–Ukraine war escalated in early 2022, at a moment when Latin American populations were still experiencing the prolonged psychosocial, economic, and institutional effects of the COVID-19 pandemic (Palomera-Chávez et al., 2021; Gutiérrez-Calderón et al., 2023; Ulloa et al., 2022). This temporal overlap is particularly relevant because the pandemic had already produced substantial mental health burden across the region, including symptoms of anxiety, depression, perceived stress, and distress related to pandemic news, uncertainty, and socioeconomic disruption (Palomera-Chávez et al., 2021; Ulloa et al., 2022). Younger individuals and women were consistently identified as groups with greater vulnerability to pandemic-related psychological distress in Latin American and international studies, while prolonged exposure to crisis-related information and reduced coping resources appeared to intensify these responses (Gato et al., 2021; Varma et al., 2021; Ulloa et al., 2022; Phalswal et al., 2023).

Latin America therefore represents a meaningful setting in which to examine how a distant geopolitical conflict may be psychologically processed in a context marked by social inequality, political instability, uneven access to mental health care, and residual distress from the pandemic period. Rather than assessing direct war trauma, the present study focuses on perceived stress related to the Russia–Ukraine war, understood as a subjective stress response to the possibility, escalation, and broader consequences of the conflict. This construct is conceptually distinct from generalized anxiety, fear alone, or post-traumatic stress disorder, although it may share cognitive and emotional pathways related to uncertainty, threat appraisal, and perceived loss of control. Accordingly, the objective of this study was to determine the association between country of residence and perceived stress related to the Russia–Ukraine war among adults from nine Latin American countries.

## Materials and methods

### Design and population

A cross-sectional analytical study was conducted across nine Latin American countries during March and April 2022, shortly after the beginning of the Russia–Ukraine war. The participating countries were Colombia, Paraguay, Peru, Bolivia, Ecuador, Mexico, Panama, Brazil, and Argentina. Data collection was performed through an online survey disseminated via social media platforms and local academic and research networks in each participating country. The target population consisted of adults aged 18 years or older who reported residing in one of the participating Latin American countries at the time of data collection and who voluntarily agreed to participate after reviewing the electronic informed consent form. Sampling was non-probabilistic and based on convenience recruitment.

The original sample size calculation indicated that at least 2,178 participants were required to detect a minimum expected difference of 3 percentage points between comparison groups, assuming 80% statistical power and a 95% confidence level. A larger number of responses was initially collected to account for incomplete questionnaires and data cleaning procedures. After applying the predefined analytical criteria, the final study sample comprised 2,463 participants from nine countries. Colombia contributed 563 participants, Paraguay 400, Peru 399, Bolivia 309, Ecuador 239, Mexico 190, Panama 167, Brazil 128, and Argentina 68. The sociodemographic composition of the final analytical sample, both overall and stratified by country, is presented in Table 1.

**Table 1:** Sociodemographic characteristics of the analytical sample by country.

Country	Sample size, *n* (%)	Sex		Age, median (IQR)	Educational level			
		Women, *n* (%)	Men, *n* (%)		Secondary or below, *n* (%)	Technical/Vocational, *n* (%)	University, *n* (%)	Postgraduate, *n* (%)
Colombia	563 (22.86)	342 (60.75)	221 (39.25)	21 (19–24)	67 (11.90)	49 (8.70)	419 (74.42)	28 (4.97)
Paraguay	400 (16.24)	245 (61.25)	155 (38.75)	24 (21–29)	31 (7.75)	16 (4.00)	302 (75.50)	51 (12.75)
Peru	399 (16.20)	223 (55.89)	176 (44.11)	24 (22–28)	26 (6.52)	18 (4.51)	318 (79.70)	37 (9.27)
Bolivia	309 (12.55)	189 (61.17)	120 (38.83)	26 (22–29)	35 (11.33)	16 (5.18)	205 (66.34)	53 (17.15)
Ecuador	239 (9.70)	176 (73.64)	63 (26.36)	23 (22–25)	29 (12.13)	2 (0.84)	199 (83.26)	9 (3.77)
Mexico	190 (7.71)	123 (64.74)	67 (35.26)	23 (21–37)	12 (6.32)	5 (2.63)	141 (74.21)	32 (16.84)
Panama	167 (6.78)	103 (61.68)	64 (38.32)	22 (19–29)	15 (8.98)	3 (1.80)	131 (78.44)	18 (10.78)
Brazil	128 (5.20)	62 (48.44)	66 (51.56)	33 (27–43)	29 (22.66)	38 (29.69)	50 (39.06)	11 (8.59)
Argentina	68 (2.76)	49 (72.06)	19 (27.94)	26.5 (23–52)	19 (27.94)	3 (4.41)	45 (66.18)	1 (1.47)
Total	2,463 (100.00)	1,512 (61.39)	951 (38.61)	23 (21–28)	263 (10.68)	150 (6.09)	1,810 (73.49)	240 (9.74)

Values are presented as *n* (%) unless otherwise specified. IQR, interquartile range.

### Variables and outcome definitions

The primary construct of interest was perceived stress related to the Russia–Ukraine war, operationalized using the War-Stress scale, a previously developed and validated Latin American instrument designed to assess psychological stress responses associated with the possibility and consequences of a distant armed conflict (Mejia et al., 2023). The scale includes nine items, each assessed using a five-point Likert response format ranging from 1, corresponding to strongly disagree, to 5, corresponding to strongly agree. Higher scores indicate greater perceived stress associated with the war context.

The nine items are conceptually organized into two subcomponents. The first subcomponent captures conflict-specific stress factors, reflecting personal perceptions of reduced control, inability to cope, and distress in the context of war. This dimension included six items related to being unable to manage personal problems, perceiving that things were not going well, being unable to cope with pending responsibilities, being unable to control difficulties in life, feeling unable to keep the situation under control, and becoming upset because of being unable to control the situation. The second subcomponent captures external stress factors, reflecting stress associated with broader political and informational elements of the conflict. This dimension included three items related to decisions made by politicians, the situation portrayed by the media, and the perception that the conflict could escalate into a larger war. The full distribution of responses for each item is shown in Table 2.

**Table 2:** Response distribution for the nine War-Stress items in the analytical sample.

War-Stress item	Strongly disagree, *n* (%)	Disagree, *n* (%)	Indifferent, *n* (%)	Agree, *n* (%)	Strongly agree, *n* (%)
Being unable to manage my personal problems in this context of war	550 (22.33)	736 (29.88)	730 (29.64)	359 (14.58)	88 (3.57)
Things are not going well for me in this context of war	543 (22.05)	710 (28.83)	723 (29.35)	387 (15.71)	100 (4.06)
Not being able to cope with everything I need to do in this context of war	553 (22.45)	756 (30.69)	704 (28.58)	361 (14.66)	89 (3.61)
Not being able to control the difficulties in my life in this context of war	544 (22.09)	773 (31.38)	701 (28.46)	362 (14.70)	83 (3.37)
Feeling unable to keep this situation under control in the context of war	557 (22.61)	739 (30.00)	677 (27.49)	394 (16.00)	96 (3.90)
Getting angry/upset because I cannot control this situation	536 (21.76)	695 (28.22)	686 (27.85)	419 (17.01)	127 (5.16)
Decisions made by politicians	427 (17.34)	455 (18.47)	524 (21.27)	697 (28.30)	360 (14.62)
The situation portrayed by the media	415 (16.85)	467 (18.96)	558 (22.66)	728 (29.56)	295 (11.98)
This situation is escalating, and a larger war is breaking out	377 (15.31)	414 (16.81)	458 (18.60)	732 (29.72)	482 (19.57)

Three continuous scores were constructed by summing item responses. The overall War-Stress score was calculated from all nine items, the conflict-specific stress score from the six conflict-specific items, and the external-factor stress score from the three external-factor items. For the main analyses, each score was categorized using its empirical upper tercile in the final analytical sample. Participants were classified as having high overall war-related stress when their total War-Stress score was 29 points or higher, corresponding to the upper empirical tercile of the observed distribution. Similarly, participants were classified as having high conflict-specific stress when their conflict-specific score was 19 points or higher, and as having high external-factor stress when their external-factor score was 13 points or higher.

These cutoffs were verified through direct inspection of the empirical tercile classification and were contrasted with the 66.7th percentile of each score distribution. The 66.7th percentile corresponded to 28 points for the total scale, 18 points for the conflict-specific subscale, and 12 points for the external-factor subscale. However, because of ties in the observed score distributions, particularly for the conflict-specific and external-factor subscales, using a simple “greater than or equal to the percentile value” rule would have resulted in substantially larger proportions of participants being classified as highly stressed than the intended upper tercile. For this reason, the empirical upper tercile definition was retained as the primary operational criterion, preserving mutually exclusive tercile-based categories and maintaining consistency with the analytical strategy used to define the outcome.

The main explanatory variable was country of residence, using Colombia as the reference category in regression models. Covariates included sex, categorized as women or men; age, measured in completed years and treated as a continuous variable in the regression analyses; and educational level, categorized as secondary education or below, technical or vocational education, university education, or postgraduate education.

### Internal consistency and structural assessment of the War-Stress scale

The internal consistency of the War-Stress instrument was assessed using Cronbach’s alpha coefficient for the total nine-item scale and for each of the two prespecified subcomponents. In the full analytical sample, reliability was excellent for the total War-Stress scale, with a Cronbach’s alpha of 0.9299. Reliability was also excellent for the conflict-specific stress factor, with an alpha of 0.9490, and high for the external-factor stress dimension, with an alpha of 0.8937. These results are summarized in Table 3.

**Table 3:** Internal consistency and structural assessment of the War-Stress scale.

Assessment	Result	Interpretation
Cronbach’s alpha—total 9-item War-Stress scale	*α* = 0.9299	Excellent internal consistency
Cronbach’s alpha—conflict-specific stress factor	*α* = 0.9490	Excellent internal consistency
Cronbach’s alpha—external stress factor	*α* = 0.8937	High internal consistency
Ordinal CFA—total analytical sample	Two-factor correlated model converged	All factor loadings were positive and statistically significant
Latent factor covariance	0.638; *p* < 0.001	Moderate-to-high positive correlation between latent factors
Country-specific ordinal CFA convergence	Converged in Paraguay, Peru, Ecuador, Panama, and Brazil	The prespecified structure was estimable in five countries
Multigroup configural ordinal CFA	No numerically stable solution was obtained	Formal cross-country configural equivalence could not be established

The ordinal confirmatory factor analysis was estimated using generalized structural equation modeling with an ordinal probit link. The global model included 2,463 participants and supported the prespecified two-factor structure. The multigroup configural model across the nine countries did not yield a stable estimation.

In response to the need to examine the performance of the instrument across participating countries, Cronbach’s alpha coefficients were also estimated separately by country for the total scale and for both subcomponents. Across countries, internal consistency remained high to excellent for the total scale, ranging from 0.9046 in Bolivia to 0.9453 in Peru. The conflict-specific factor showed consistently excellent reliability across countries, with alpha coefficients ranging from 0.9342 in Bolivia to 0.9701 in Brazil. The external-factor dimension also showed adequate to excellent reliability, with alpha values ranging from 0.8160 in Brazil to 0.9266 in Argentina. Country-specific reliability estimates are reported in Supplementary Table S1.

To further evaluate the structural validity of the instrument, an ordinal confirmatory factor analysis was estimated for the full analytical sample using a generalized structural equation modeling framework with an ordinal probit link, treating the nine Likert items as ordinal indicators. The prespecified model included two correlated latent factors. The first latent factor represented conflict-specific stress and was defined by the six items addressing personal perceptions of coping difficulties and lack of control in the context of war. The second latent factor represented external-factor stress and was defined by the three items addressing political decisions, media portrayals, and the possibility of a broader war.

The global two-factor ordinal confirmatory factor analysis converged successfully. All factor loadings were positive and statistically significant, supporting the prespecified structure of the War-Stress instrument in the pooled multinational sample. The latent covariance between the two dimensions was 0.638 and was statistically significant, indicating a moderate-to-high positive relationship between conflict-specific and external stress responses. The global psychometric summary of the ordinal confirmatory factor analysis is included in Table 3.

As an exploratory country-level structural assessment, the same ordinal two-factor confirmatory factor analysis model was estimated separately within each country. Country-specific models converged in Paraguay, Peru, Ecuador, Panama, and Brazil, and in all convergent models the factor loadings were positive and statistically significant. The estimated covariance between latent dimensions ranged from 0.567 in Paraguay to 0.769 in Peru, suggesting that the two dimensions of war-related stress remained positively associated across the countries in which stable solutions were obtained. In contrast, models for Colombia, Bolivia, Mexico, and Argentina did not achieve stable convergence under the prespecified ordinal generalized structural equation modeling specification. This lack of convergence may reflect country-specific sample size limitations, unequal response distributions across ordinal categories, and numerical instability associated with estimating multiple thresholds and latent parameters within smaller or distributionally constrained strata. These country-level findings are reported in Supplementary Table S4.

A multigroup configural ordinal confirmatory factor analysis across all nine countries was also attempted to evaluate whether the same general latent structure could be simultaneously estimated across countries. However, this model did not yield a numerically stable solution and failed to converge. Therefore, formal cross-country configural equivalence could not be established within the present analytical framework. Accordingly, the ordinal confirmatory factor analysis findings were interpreted as supporting the overall two-factor structure in the pooled analytical sample, while country-level structural equivalence was considered exploratory and not conclusive.

### Procedures and ethical considerations

The study protocol was reviewed and approved by the Ethics Committee of Norbert Wiener University, Peru, through Official Letter No. 1647-2022. The study was conducted in accordance with the ethical principles of the Declaration of Helsinki. At the beginning of the online questionnaire, all participants received information regarding the objectives of the study, the voluntary nature of participation, confidentiality protections, and the possibility of withdrawing from the survey at any moment before submission. Only individuals who provided electronic informed consent were allowed to proceed to the questionnaire. No personally identifying information was collected in the analytical dataset.

Data were collected using the Google Forms platform, which was selected to facilitate remote participation and preserve anonymity during an ongoing period of pandemic-related public health restrictions. After data collection, the information was exported to Microsoft Excel for initial screening and data quality review. The database was subsequently processed according to the predefined eligibility and cleaning criteria and then imported into Stata statistical software for descriptive, psychometric, and inferential analyses.

### Statistical analysis

A descriptive analysis was first conducted for the final analytical sample. Categorical variables were summarized using absolute frequencies and percentages. Age was described using the median and interquartile range because of its non-normal distribution. Sociodemographic characteristics were summarized overall and by country, as shown in Table 1. The distribution of responses to each War-Stress item was described using absolute and relative frequencies, as presented in Table 2.

The reliability and structural properties of the scale were examined through Cronbach’s alpha coefficients and ordinal confirmatory factor analyses, as detailed above. The global reliability and confirmatory factor analysis results are summarized in Table 3, whereas country-specific reliability estimates and exploratory country-level confirmatory factor analysis findings are reported in Supplementary Tables S1, S4, respectively.

For the association analyses, three binary outcomes were evaluated: high overall war-related stress, high conflict-specific stress, and high external-factor stress. Adjusted prevalence ratios were estimated using generalized linear models with Poisson family, log link function, and robust variance estimators. Country of residence was the main explanatory variable, and all multivariable models were adjusted for sex, age, and educational level. Adjusted prevalence ratios are reported with their corresponding 95% confidence intervals and *p*-values. Statistical significance was defined as *p* < 0.05.

To address the reviewer’s concern regarding unequal country-specific sample sizes, particularly the small sample from Argentina, two sensitivity analyses were performed. In the first sensitivity analysis, all adjusted models were re-estimated after excluding Argentina from the analytical sample. In the second sensitivity analysis, models were restricted to countries with at least 200 participants, namely Colombia, Paraguay, Peru, Bolivia, and Ecuador. The stability of key estimates across the main and sensitivity models was examined and is summarized in Table 5.

**Table 5:** Sensitivity analyses for the main adjusted associations.

Key association	Main model: 9 countries	Sensitivity analysis 1: excluding Argentina	Sensitivity analysis 2: countries with *n* ≥ 200
Overall high stress			
Men vs. women	0.63 (0.55–0.73)	0.62 (0.54–0.72)	0.62 (0.53–0.72)
Argentina vs. Colombia	1.73 (1.33–2.25)	Not applicable	Not applicable
Peru vs. Colombia	0.90 (0.73–1.10)	0.90 (0.74–1.11)	0.89 (0.73–1.10)
Conflict-specific high stress			
Men vs. women	0.66 (0.57–0.77)	0.66 (0.56–0.78)	0.65 (0.55–0.78)
Argentina vs. Colombia	1.58 (1.13–2.21)	Not applicable	Not applicable
Brazil vs. Colombia	0.64 (0.40–1.01)	0.65 (0.41–1.03)	Not applicable
External-factor high stress			
Men vs. women	0.65 (0.54–0.78)	0.61 (0.50–0.75)	0.68 (0.55–0.84)
Peru vs. Colombia	0.52 (0.38–0.73)	0.54 (0.38–0.75)	0.52 (0.37–0.73)
Brazil vs. Colombia	0.50 (0.28–0.90)	0.56 (0.31–1.01)	Not applicable

Values are adjusted prevalence ratios (95% confidence intervals). Sensitivity analysis 1 excluded Argentina because it had the smallest country-specific sample size. Sensitivity analysis 2 retained only countries with at least 200 participants.

To explore whether the association between sex and perceived stress differed according to age or educational attainment, interaction terms were added to the adjusted Poisson models. Specifically, models were estimated for the interaction between sex and age and for the interaction between sex and educational level. Global Wald tests were used to evaluate statistical evidence for interaction. When sex-by-age interaction terms were statistically significant or borderline significant, predictive margins were estimated by sex at selected age values of 18, 25, 35, 45, 55, and 65 years to facilitate substantive interpretation of the interaction pattern. The full interaction model results are summarized in Supplementary Tables S2, S3.

All statistical analyses were performed using Stata statistical software.

## Results

### Characteristics of the analytical sample

The final analytical sample included 2,463 participants from nine Latin American countries. Colombia contributed the largest proportion of respondents, with 563 participants corresponding to 22.86% of the sample, followed by Paraguay with 400 participants, Peru with 399, Bolivia with 309, Ecuador with 239, Mexico with 190, Panama with 167, Brazil with 128, and Argentina with 68 participants. The complete distribution of the analytical sample by country is presented in Table 1.

Overall, 61.39% of participants were women and 38.61% were men. The median age in the full sample was 23 years, with an interquartile range of 21 to 28 years. Country-specific age distributions showed a younger median age in Colombia, with a median of 21 years and an interquartile range of 19 to 24, and in Panama, with a median of 22 years and an interquartile range of 19 to 29. In contrast, Brazil and Argentina showed comparatively older age profiles, with median ages of 33 years and 26.5 years, respectively. Regarding educational attainment, 73.49% of participants reported university education, 10.68% secondary education or below, 6.09% technical or vocational education, and 9.74% postgraduate studies. The country-specific sociodemographic composition of the sample is detailed in Table 1.

### Distribution of War-Stress item responses

The distribution of responses across the nine War-Stress items revealed two distinct response patterns. Items reflecting personal perceived control and coping difficulties in the context of war tended to show higher frequencies in the categories of disagreement or indifference. In contrast, items related to broader external threats and uncertainty showed markedly higher levels of agreement.

The item with the highest endorsement of stress was the statement that the situation was escalating and that a larger war could break out. For this item, 29.72% of respondents agreed and 19.57% strongly agreed. High endorsement was also observed for stress related to the situation portrayed by the media, with 29.56% agreeing and 11.98% strongly agreeing, as well as for stress related to decisions made by politicians, with 28.30% agreeing and 14.62% strongly agreeing. These findings indicate that stress associated with the war was especially pronounced when participants evaluated geopolitical uncertainty, media exposure, and the possibility of escalation beyond the immediate Russia–Ukraine context.

By contrast, the six items representing conflict-specific personal stress responses had lower proportions of strong agreement, generally ranging from 3.37 to 5.16%. Nevertheless, substantial proportions of respondents selected the indifferent category across these items, suggesting that the war context generated heterogeneous psychological reactions, with greater endorsement of macro-level and externally mediated stressors than of personally internalized coping difficulties. The complete distribution of responses for each War-Stress item is presented in Table 2.

### Classification of high perceived stress

Using the empirical upper tercile criterion, 769 participants, corresponding to 31.22% of the analytical sample, were classified as having high overall war-related stress. This classification corresponded to a total War-Stress score of 29 points or higher. For the conflict-specific subcomponent, 613 participants, representing 24.89% of the sample, were classified as having high conflict-specific stress, corresponding to a score of 19 points or higher. For the external-factor subcomponent, 465 participants, representing 18.88% of the sample, were classified as having high external-factor stress, corresponding to a score of 13 points or higher.

The proportion of participants classified in the empirical upper tercile varied across countries. High overall stress was most frequent in Argentina, where 54.41% of respondents were classified in the upper tercile, followed by Ecuador with 36.82% and Panama with 34.73%. The lowest proportion of high overall stress was observed in Brazil, where 20.31% of participants met the upper-tercile criterion. High conflict-specific stress was also most frequent in Argentina, with 39.71%, followed by Ecuador with 31.80%, Bolivia with 28.16%, and Panama with 28.14%. High external-factor stress was especially elevated in Argentina, where 60.29% of participants were classified in the upper tercile, whereas the lowest proportions were identified in Brazil, with 8.59%, and Peru, with 10.28%. These descriptive country-level differences provide important context for the adjusted analyses presented later in the manuscript.

### Internal consistency and structural assessment of the War-Stress scale

The War-Stress instrument showed strong psychometric performance in the pooled analytical sample. Cronbach’s alpha coefficient was 0.9299 for the total nine-item scale, indicating excellent internal consistency. Reliability was also excellent for the conflict-specific stress factor, with an alpha coefficient of 0.9490, and high for the external-factor stress dimension, with an alpha coefficient of 0.8937. These findings indicate that the items displayed a high degree of internal coherence both at the level of the overall construct and within each of the two conceptual subdimensions. The global reliability results are presented in Table 3.

Country-specific reliability estimates were consistently high. The total War-Stress scale showed Cronbach’s alpha values above 0.90 in all nine countries, ranging from 0.9046 in Bolivia to 0.9453 in Peru. The conflict-specific factor demonstrated very high consistency across countries, with alpha coefficients between 0.9342 and 0.9701. The external-factor subscale also performed adequately across all countries, with alpha coefficients ranging from 0.8160 in Brazil to 0.9266 in Argentina. These findings support the internal consistency of the scale within each participating national subgroup, although they do not in themselves establish formal measurement invariance across countries. The complete country-specific reliability profile is provided in Supplementary Table S1.

The ordinal confirmatory factor analysis conducted in the full analytical sample supported the prespecified two-factor correlated structure. The model converged successfully and included all 2,463 participants. All item loadings were positive and statistically significant. Within the conflict-specific stress factor, the highest coefficients were observed for the items related to inability to cope with responsibilities in the context of war, inability to control difficulties in life, and feeling unable to manage the situation. Within the external-factor stress dimension, all three indicators loaded significantly on the latent construct, with substantial contributions from stress related to the situation portrayed by the media and to decisions made by politicians. The covariance between the two latent factors was 0.638 and statistically significant, supporting a meaningful positive association between conflict-specific stress and external-factor stress. A summary of these global psychometric findings is presented in Table 3.

Country-specific ordinal confirmatory factor analysis models converged in Paraguay, Peru, Ecuador, Panama, and Brazil. In these five countries, all factor loadings were positive and statistically significant, preserving the expected direction of association between the observed ordinal indicators and the two latent stress dimensions. The latent factor covariance ranged from 0.567 in Paraguay to 0.769 in Peru, suggesting that the relationship between the conflict-specific and external-factor dimensions remained positive across the countries in which stable models were obtained. In contrast, the models estimated for Colombia, Bolivia, Mexico, and Argentina did not converge under the prespecified ordinal generalized structural equation modeling approach. The multigroup configural model including all nine countries also failed to reach a stable solution. Therefore, the available evidence supports the two-factor structure at the pooled sample level and provides partial exploratory support at the country level, but does not permit strong claims regarding full cross-country structural equivalence. Detailed country-level model information is presented in Supplementary Table S4.

### Main adjusted associations with high perceived stress

In the multivariable model for high overall war-related stress, adjusted for sex, age, and educational level, participants from Argentina showed a significantly higher prevalence of high stress compared with participants from Colombia. The adjusted prevalence ratio was 1.73, with a 95% confidence interval from 1.33 to 2.25 and a *p*-value below 0.001. No statistically significant differences were identified for Paraguay, Peru, Bolivia, Ecuador, Mexico, Panama, or Brazil relative to Colombia. Men had a substantially lower prevalence of high overall stress than women, with an adjusted prevalence ratio of 0.63, a 95% confidence interval from 0.55 to 0.73, and a *p*-value below 0.001. Age was not statistically associated with high overall stress in the main adjusted model, with an adjusted prevalence ratio per additional year of 0.994 and a *p*-value of 0.104. Participants with postgraduate education showed a lower prevalence of high overall stress compared with those with secondary education or below, with an adjusted prevalence ratio of 0.74, a 95% confidence interval from 0.56 to 0.99, and a *p*-value of 0.040. The full adjusted results for the three primary outcomes are presented in Table 4.

**Table 4:** Main adjusted associations with high perceived stress related to the Russia–Ukraine war.

Predictor	Adjusted prevalence ratio (95% CI)	*p*-value
Overall high war-related stress		
Paraguay vs. Colombia	1.05 (0.87–1.27)	0.578
Peru vs. Colombia	0.90 (0.73–1.10)	0.297
Bolivia vs. Colombia	1.04 (0.85–1.27)	0.716
Ecuador vs. Colombia	1.12 (0.91–1.37)	0.298
Mexico vs. Colombia	0.94 (0.73–1.22)	0.655
Panama vs. Colombia	1.13 (0.89–1.44)	0.307
Brazil vs. Colombia	0.75 (0.52–1.09)	0.128
Argentina vs. Colombia	**1.73 (1.33–2.25)**	**<0.001**
Men vs. women	**0.63 (0.55–0.73)**	**<0.001**
Conflict-specific high stress		
Paraguay vs. Colombia	1.05 (0.84–1.32)	0.646
Peru vs. Colombia	0.93 (0.73–1.18)	0.553
Bolivia vs. Colombia	1.21 (0.96–1.52)	0.107
Ecuador vs. Colombia	1.26 (0.99–1.60)	0.058
Mexico vs. Colombia	0.90 (0.66–1.24)	0.530
Panama vs. Colombia	1.20 (0.90–1.59)	0.218
Brazil vs. Colombia	0.64 (0.40–1.01)	0.054
Argentina vs. Colombia	**1.58 (1.13–2.21)**	**0.008**
Men vs. women	**0.66 (0.57–0.77)**	**<0.001**
External-factor high stress		
Paraguay vs. Colombia	1.03 (0.80–1.33)	0.814
Peru vs. Colombia	**0.52 (0.38–0.73)**	**<0.001**
Bolivia vs. Colombia	0.94 (0.71–1.24)	0.653
Ecuador vs. Colombia	0.91 (0.67–1.23)	0.548
Mexico vs. Colombia	1.01 (0.73–1.41)	0.936
Panama vs. Colombia	0.79 (0.54–1.15)	0.225
Brazil vs. Colombia	**0.50 (0.28–0.90)**	**0.020**
Argentina vs. Colombia	**3.03 (2.30–3.99)**	**<0.001**
Men vs. women	**0.65 (0.54–0.78)**	**<0.001**

Adjusted prevalence ratios were estimated using Poisson regression with a log link and robust variance. All models included country of residence, sex, age, and educational level. Reference categories were Colombia for country, women for sex, and secondary education or below for education. Bold values indicate statistically significant associations at *p* < 0.05.

For high conflict-specific stress, Argentina again exhibited a significantly higher prevalence compared with Colombia. The adjusted prevalence ratio was 1.58, with a 95% confidence interval from 1.13 to 2.21 and a *p*-value of 0.008. Ecuador showed a borderline association with higher conflict-specific stress, with an adjusted prevalence ratio of 1.26 and a *p*-value of 0.058, whereas Brazil showed a borderline lower prevalence, with an adjusted prevalence ratio of 0.64 and a *p*-value of 0.054. Men had a lower prevalence of high conflict-specific stress than women, with an adjusted prevalence ratio of 0.66, a 95% confidence interval from 0.57 to 0.77, and a *p*-value below 0.001. Educational attainment was also associated with this outcome, as participants with postgraduate education showed lower prevalence compared with the reference group, with an adjusted prevalence ratio of 0.67, a 95% confidence interval from 0.48 to 0.94, and a *p*-value of 0.020. These findings are also shown in Table 4.

In the model for high external-factor stress, the strongest association was observed for Argentina, where the adjusted prevalence was more than three times that of Colombia. The adjusted prevalence ratio was 3.03, with a 95% confidence interval from 2.30 to 3.99 and a *p*-value below 0.001. In contrast, participants from Peru had a significantly lower prevalence of high external-factor stress, with an adjusted prevalence ratio of 0.52, a 95% confidence interval from 0.38 to 0.73, and a *p*-value below 0.001. Participants from Brazil also showed a lower prevalence, with an adjusted prevalence ratio of 0.50, a 95% confidence interval from 0.28 to 0.90, and a *p*-value of 0.020. Men again showed lower prevalence than women, with an adjusted prevalence ratio of 0.65, a 95% confidence interval from 0.54 to 0.78, and a *p*-value below 0.001. Increasing age was associated with a modest but statistically significant reduction in high external-factor stress, with an adjusted prevalence ratio per additional year of 0.988, a 95% confidence interval from 0.978 to 0.998, and a *p*-value of 0.015. No statistically significant adjusted differences were observed for educational attainment in this model. Complete estimates are reported in Table 4.

### Sensitivity analyses addressing unequal country sample sizes

Because Argentina had the smallest country-specific sample size and showed the most extreme adjusted associations in the primary models, two sensitivity analyses were conducted to evaluate the robustness of the main findings. In the first sensitivity analysis, all adjusted models were re-estimated after excluding Argentina, leaving 2,395 participants. The associations observed for sex remained stable across all outcomes. Men continued to exhibit a lower prevalence of high overall stress, high conflict-specific stress, and high external-factor stress. Specifically, the adjusted prevalence ratio for men was 0.62 in the overall stress model, 0.66 in the conflict-specific stress model, and 0.61 in the external-factor stress model. Peru continued to show lower external-factor stress, with an adjusted prevalence ratio of 0.54, while the lower prevalence observed in Brazil remained directionally consistent, although the confidence interval included the null value after Argentina was excluded.

In the second sensitivity analysis, models were restricted to countries with at least 200 participants, namely Colombia, Paraguay, Peru, Bolivia, and Ecuador, yielding an analytical subsample of 1,910 participants. In these restricted models, the association between male sex and lower perceived stress remained consistent across all three outcomes. Men had lower prevalence of high overall stress, with an adjusted prevalence ratio of 0.62, lower prevalence of high conflict-specific stress, with an adjusted prevalence ratio of 0.65, and lower prevalence of high external-factor stress, with an adjusted prevalence ratio of 0.68. Peru also remained associated with lower prevalence of high external-factor stress, with an adjusted prevalence ratio of 0.52. These sensitivity analyses indicate that the principal associations involving sex and Peru’s lower external-factor stress were robust to the exclusion of Argentina and to restriction of the analysis to countries with larger sample sizes. The comparative results from the primary and sensitivity models are summarized in Table 5.

### Interaction analyses

To explore whether the relationship between sex and high perceived stress varied according to age, adjusted models including sex-by-age interaction terms were estimated. A statistically significant interaction was observed for high overall war-related stress. The interaction prevalence ratio was 1.017, with a 95% confidence interval from 1.004 to 1.030 and a *p*-value of 0.010. The global Wald test for the interaction was also statistically significant, with *p* = 0.010. This interaction pattern suggested that the difference in predicted prevalence between women and men narrowed progressively with increasing age. At 18 years of age, the predicted probability of high overall stress was 40.1% among women and 21.9% among men. At 25 years, the corresponding predicted probabilities were 36.6 and 22.5%, respectively. By 45 years, the values had become closer, reaching 28.4% among women and 24.4% among men. At 55 years, the predicted probabilities were nearly equivalent, at 25.0% among women and 25.4% among men. These findings are summarized in Supplementary Table S2.

A borderline significant sex-by-age interaction was identified for high conflict-specific stress. The interaction prevalence ratio was 1.015, with a 95% confidence interval from 1.000 to 1.030 and a *p*-value of 0.050. The global Wald test yielded the same significance level. As in the overall stress model, sex differences were more pronounced among younger participants and became smaller at older ages. At 18 years, the predicted probability of high conflict-specific stress was 30.5% among women and 17.6% among men. At 25 years, these predicted probabilities were 28.7 and 18.4%, respectively. By 55 years, the predicted prevalence was nearly identical between women and men, at 22.3 and 22.2%, respectively. These results are also included in Supplementary Table S2.

No statistically significant sex-by-age interaction was found for high external-factor stress. The interaction prevalence ratio was 1.010, with a 95% confidence interval from 0.992 to 1.028 and a *p*-value of 0.271. This finding indicates that there was no clear evidence that the association between sex and external-factor stress varied across age in the present sample.

Models examining sex-by-educational-level interactions showed no statistically significant global interaction for any of the three outcomes. The global Wald test yielded *p* = 0.134 for high overall stress, *p* = 0.348 for high conflict-specific stress, and *p* = 0.777 for high external-factor stress. Although some individual interaction contrasts were suggestive, the global tests did not support a consistent pattern of effect modification by educational level. These results are presented in Supplementary Table S3.

## Discussion

This multicountry study found that perceived stress related to the Russia–Ukraine war varied across Latin American countries and was shaped by both contextual and sociodemographic factors. In the primary adjusted analyses, Argentina showed the highest prevalence of overall and external-factor war-related stress, whereas Peru and Brazil showed lower prevalence of high external-factor stress. Men consistently exhibited lower prevalence of high perceived stress across the three outcomes examined. In addition, the interaction analyses indicated that sex differences in overall stress were more pronounced at younger ages and progressively narrowed with increasing age. These principal patterns remained broadly consistent in sensitivity analyses excluding Argentina and in models restricted to countries with larger sample sizes, supporting the stability of several core findings while reinforcing the need for caution when interpreting country-specific estimates derived from smaller strata.

The results contribute to the growing literature showing that the psychological impact of war is not limited to populations directly exposed to combat, displacement, bereavement, or destruction. Contemporary crises can also generate distress in geographically distant populations through mediated exposure, anticipatory threat, and perceptions that the conflict may produce wider political, economic, or humanitarian consequences. Prior studies conducted during the Russia–Ukraine war have documented stress, anxiety, depressive symptoms, and post-traumatic manifestations in populations located outside the direct combat zone, including individuals in Poland, Taiwan, Finland, and other European settings (Chudzicka-Czupała et al., 2023; Celuch et al., 2024; Scharbert et al., 2024). Evidence from other conflict-related contexts and diaspora populations further supports the existence of vicarious psychological burden among people who are physically distant from violence but emotionally or informationally connected to it (Gesesew et al., 2025).

In the present study, the War-Stress items with the highest endorsement were precisely those related to external and anticipatory threats: the perception that the conflict could escalate into a larger war, stress associated with media portrayals, and concern about decisions made by political actors. This response pattern suggests that the psychological burden captured in this Latin American sample was driven less by direct personal consequences of the war and more by appraisal of uncertainty, lack of control, and perceived global threat. This interpretation is consistent with stress appraisal models, in which distress emerges when individuals evaluate an event as highly consequential, unpredictable, and difficult to manage with available coping resources (Taha et al., 2014; Spătaru et al., 2024). It is also consistent with research showing that media exposure during collective crises can amplify acute stress, anxiety, and depressive symptoms, particularly when individuals experience high intolerance of uncertainty or perceive limited support and control (Phalswal et al., 2023; Kesner et al., 2025).

The timing of data collection is essential for interpreting these findings. The survey was conducted only weeks after the escalation of the Russia–Ukraine war, while Latin American societies were still confronting the consequences of the COVID-19 pandemic. This overlap of crises may have intensified psychological receptivity to a new global threat. Studies have shown that anxieties related to COVID-19 and the war in Ukraine can accumulate and jointly contribute to perceived stress and psychological distress (Celuch et al., 2024). In Latin America, the pandemic had already produced substantial mental health burden across multiple countries, with elevated stress, anxiety, and depressive symptoms in different population groups, as well as persistent concerns related to infection, economic instability, and social disruption (Bedoya Cardona et al., 2021; Palomera-Chávez et al., 2021). Within this context, the Russia–Ukraine war may have been experienced not as an isolated foreign event, but as an additional layer of uncertainty superimposed on an already strained psychosocial environment.

The high adjusted prevalence ratios observed for Argentina require particular caution. Although Argentina showed the greatest prevalence of overall and external-factor war-related stress in the main models, it also had the smallest country-specific sample. For this reason, the study incorporated sensitivity analyses excluding Argentina and restricting the analysis to countries with at least 200 participants. These analyses confirmed the stability of the main findings related to sex and Peru’s lower external-factor stress, while also underscoring that the Argentina-specific estimates should be considered suggestive rather than definitive. The Argentine pattern may reflect heightened sensitivity to broader external uncertainty, local socioeconomic stressors, characteristics of the recruited participants, or a combination of these mechanisms. However, the present data do not permit a causal or nationally representative explanation, and future studies with larger and more balanced country samples will be necessary to determine whether this finding is reproducible.

The lower prevalence of high external-factor stress observed in Peru also warrants a nuanced interpretation. Rather than indicating an absence of distress, this result may suggest that war-related external concerns were comparatively less salient among Peruvian respondents after accounting for sex, age, and educational level. One possible explanation is that populations already exposed to sustained domestic political instability, pandemic-related hardship, and chronic socioeconomic stress may perceive an external geopolitical conflict differently from populations in other settings. However, this remains a hypothesis rather than a demonstrable mechanism in the present study. A similarly cautious reading is required for Brazil, where lower external-factor stress was observed in the primary model but showed weaker stability in sensitivity analysis. These country-level patterns should therefore be interpreted as comparative findings within a non-probabilistic multicountry sample, not as nationally representative estimates of collective stress in Latin America.

Sex differences were among the most consistent findings of this study. Men showed lower prevalence of high perceived stress across the overall, conflict-specific, and external-factor outcomes. This pattern aligns with prior evidence indicating that women often report greater emotional distress in response to large-scale crises, including pandemics, disasters, and war-related threats (Palomera-Chávez et al., 2021; Ulloa et al., 2022; Chudzicka-Czupała et al., 2023) mechanisms may contribute to these differences, including unequal caregiving burdens, greater exposure to emotionally salient information, differential coping styles, and higher vulnerability to uncertainty-related distress. In Latin American youth, women reported greater stress related to pandemic news, infection risk, and socioeconomic consequences during COVID-19, reinforcing the plausibility of a sex-related pattern in responses to subsequent global crises (Ulloa et al., 2022).

The interaction analyses extended this interpretation by showing that sex differences in overall war-related stress were especially pronounced at younger ages and gradually narrowed with increasing age. A similar, although borderline, pattern was observed for conflict-specific stress. These findings are compatible with evidence that younger individuals tend to be more psychologically vulnerable during collective crises and may be more exposed to social media, crisis-related news, and uncertain future expectations (Varma et al., 2021; Ulloa et al., 2022; Phalswal et al., 2023). The combined pattern identified here suggests that younger women may represent a particularly sensitive subgroup in the context of indirect or mediated geopolitical stress. Although this finding requires confirmation in future studies, it provides a meaningful direction for research on effect modification in crisis-related mental health.

The psychometric findings also strengthen the interpretability of the substantive results. The War-Stress scale demonstrated excellent internal consistency in the pooled analytical sample and high-to-excellent reliability across all participating countries. The ordinal confirmatory factor analysis supported the prespecified two-factor structure in the overall sample, with significant positive loadings and a meaningful covariance between the conflict-specific and external-factor dimensions. Although country-specific confirmatory models did not converge in all settings and the multigroup configural model was not numerically stable, the available results provide support for using the scale as a coherent measure of war-related perceived stress in the pooled sample while appropriately avoiding strong claims of full cross-country measurement equivalence. This distinction is important because it allows substantive interpretation of the overall findings without overstating the psychometric comparability of country-specific estimates.

Taken together, these findings support the broader public health relevance of studying indirect psychological responses to distant geopolitical conflicts. In highly interconnected information environments, wars may produce distress not only through direct exposure but also through media amplification, anticipatory threat, uncertainty regarding escalation, and concern about political or economic repercussions. For Latin American populations emerging from the psychosocial burden of COVID-19, these mechanisms may be particularly salient. Future research should use longitudinal and more representative designs, incorporate direct measures of media exposure, coping strategies, perceived control, uncertainty tolerance, and pandemic-related residual stress, and further assess the measurement invariance of war-related stress instruments across countries with larger and more balanced national samples.

## Conclusion

Perceived stress related to the Russia–Ukraine war varied across adults from the nine Latin American countries included in this study. Argentina showed a higher prevalence of overall war-related stress, conflict-specific stress, and external-factor stress compared with Colombia, although these country-specific estimates should be interpreted cautiously because Argentina contributed the smallest national sample. In contrast, Peru and Brazil showed lower prevalence of high external-factor stress in the primary adjusted model. Men consistently exhibited lower prevalence of high perceived stress across all three outcomes, and sex differences in overall stress were more pronounced at younger ages. These findings suggest that distant geopolitical conflicts may generate measurable psychological responses in populations outside the immediate conflict zone, particularly through anticipatory uncertainty, perceived escalation, and externally mediated threat. The results underscore the importance of considering broader social vulnerability, post-pandemic distress, and differential sociodemographic susceptibility when examining the mental health consequences of global crises in Latin America.

## Limitations and future research directions

This study should be interpreted in light of several limitations. First, participants were recruited through non-probabilistic convenience sampling and an online survey, which may have favored the participation of individuals with greater internet access, higher digital engagement, or stronger interest in the topic. Consequently, the findings should not be interpreted as nationally representative prevalence estimates for the participating countries. Second, country-specific sample sizes were unequal, with particularly small samples in Argentina, Brazil, and Panama. Although sensitivity analyses were conducted to evaluate the robustness of the main findings, estimates for countries with smaller subsamples should be interpreted with caution and considered exploratory. Third, the cross-sectional design precludes causal inference and does not allow determination of whether perceived war-related stress preceded, followed, or interacted with other psychological conditions or contextual stressors present during the post-pandemic period. Fourth, the study did not directly measure potentially relevant explanatory mechanisms such as intensity of media exposure, perceived economic threat, coping strategies, intolerance of uncertainty, prior mental health conditions, or residual COVID-19-related distress. These unmeasured factors may have contributed to the observed associations. Fifth, although the War-Stress scale showed excellent internal consistency and the pooled ordinal confirmatory factor analysis supported its two-factor structure, country-specific confirmatory models did not converge in all settings and formal multigroup configural equivalence could not be established. Therefore, cross-country psychometric comparability should be interpreted cautiously.

Future research should use longitudinal designs and more balanced, probabilistic, or nationally stratified samples to confirm the country-level patterns observed here. Subsequent studies should also incorporate direct measures of media consumption, appraisal of geopolitical threat, perceived control, coping resources, and ongoing pandemic-related stress in order to clarify the mechanisms linking distant conflicts with psychological distress. Larger multicountry samples would additionally permit more rigorous measurement invariance testing of the War-Stress scale and a more precise evaluation of heterogeneity across Latin American contexts. Finally, the observed sex-by-age pattern warrants further examination, particularly to determine whether younger women constitute a subgroup with greater vulnerability to indirect psychological effects of global geopolitical crises.

## Data Availability

The raw data can be made available by the authors upon reasonable request, without undue reservation.
