# Supplementary material for: Perceived stress related to the Russia–Ukraine war in nine Latin American countries
Source: Front Psychol. 2026 Jun 16;17:1845161. doi: 10.3389/fpsyg.2026.1845161 (PMC13315226; doi:10.3389/fpsyg.2026.1845161)
Supplement: Supplementary file 1 [file Data_Sheet_1.PDF]

Supplementary Tables

Supplementary Table S1. Cronbach's alpha coefficients for the War-Stress scale and its two factors by country

Country	Total 9-item scale	Conflict-specific factor	External factor
Colombia	0.9233	0.9416	0.9084
Paraguay	0.9188	0.9469	0.8784
Peru	0.9453	0.9509	0.9189
Bolivia	0.9046	0.9342	0.8231
Ecuador	0.9452	0.9538	0.9116
Mexico	0.9363	0.9610	0.8910
Panama	0.9396	0.9644	0.9026
Brazil	0.9381	0.9701	0.8160
Argentina	0.9116	0.9374	0.9266

Cronbach's alpha values indicate high to excellent internal consistency across the overall scale and both prespecified factors in all participating countries.

Supplementary Table S2. Interaction between sex and age in adjusted Poisson regression models

Outcome	Interaction term: men × age, aPR (95% CI)	p-value	Wald test for interaction
**Overall high stress**	**1.017 (1.004–1.030)**	**0.010**	**$\chi^2(1) = 6.64$; p = 0.010**
**Conflict-specific high stress**	**1.015 (1.000–1.030)**	**0.050**	**$\chi^2(1) = 3.84$; p = 0.050**
External-factor high stress	1.010 (0.992–1.028)	0.271	$\chi^2(1) = 1.21$; p = 0.271

Models were adjusted for country of residence and educational level. The interaction between sex and age was statistically significant for overall high stress and borderline for conflict-specific high stress.

Supplementary Table S3. Global tests for the interaction between sex and educational level

Outcome	Wald test	p-value	Interpretation
Overall high stress	$\chi^2(3) = 5.58$	0.134	No statistically significant interaction

Conflict-specific high stress	$\chi^2(3) = 3.30$	0.348	No statistically significant interaction
External-factor high stress	$\chi^2(3) = 1.10$	0.777	No statistically significant interaction

All interaction models were adjusted for country of residence and age. No statistically significant sex-by-education interaction was identified for any stress outcome.

Supplementary Table S4. Country-specific ordinal confirmatory factor analysis of the War-Stress scale

Two-factor correlated ordinal CFA model estimated separately within each participating country

Country	n	Model status	Latent factor covariance	95% CI	p-value	Log likelihood	AIC	BIC
Colombia	563	Did not converge	—	—	—	—	—	—
Paraguay	400	Converged	0.567	0.487 – 0.646	<0.001	-3935.881	7963.763	8147.370
Peru	399	Converged	0.769	0.720 – 0.819	<0.001	-3593.823	7279.647	7463.139
Bolivia	309	Did not converge	—	—	—	—	—	—
Ecuador	239	Converged	0.731	0.660 – 0.802	<0.001	-2270.790	4633.579	4793.497
Mexico	190	Did not converge	—	—	—	—	—	—
Panama	167	Converged	0.597	0.480 – 0.713	<0.001	-1448.711	2989.421	3132.849
Brazil	128	Converged	0.683	0.563 – 0.803	<0.001	-1055.647	2203.295	2334.488
Argentina	68	Did not converge	—	—	—	—	—	—

Supplementary Table S4. Country-specific ordinal confirmatory factor analysis of the War-Stress scale

Two-factor correlated ordinal CFA model estimated separately within each participating country

Country	n	Model status	Latent factor covariance	95% CI	p-value	Log likelihood	AIC	BIC

The ordinal confirmatory factor analysis was estimated using generalized structural equation modeling with an ordinal probit link. The prespecified model included two correlated latent factors: conflict-specific stress and external-factor stress. In countries with convergent models, all factor loadings were positive and statistically significant.

Models for Colombia, Bolivia, Mexico, and Argentina did not achieve numerically stable convergence under the prespecified country-specific ordinal GSEM specification; therefore, likelihood-based indices and latent covariance estimates are not reported for those countries.
